# Prevalence and patterns of cigarette smoking before and during early and late pregnancy according to maternal characteristics: the first national data based on the 2003 birth certificate revision, United States, 2016

**DOI:** 10.1186/s12978-019-0807-5

**Published:** 2019-09-13

**Authors:** Anthony J. Kondracki

**Affiliations:** University of Maryland, School of Public Health, Maternal and Child Health, 4200 Valley Drive, College Park, MD 20742 USA

**Keywords:** Pregnancy, Prevalence, Patterns, Smoking status, Cessation

## Abstract

**Background:**

The objective of this study was to examine the prevalence of smoking by intensity status before pregnancy and during early (first and second trimester) and late (third trimester) pregnancy according to race/ethnicity, age, and educational attainment of women who gave birth in the United States in 2016.

**Methods:**

This cross-sectional study was based on the 2016 National Center for Health Statistics (NCHS) Natality File of 3,956,112 live births, the first year that it became 100% nationally representative. Self-reported smoking data were used to create new seven smoking intensity status categories to capture natural variability in smoking patterns during pregnancy and to identify maternal smokers by race/ethnicity, age, and educational attainment. The risk of smoking at low and high intensity in early pregnancy was estimated in multivariable logistic regression analyses.

**Results:**

Nearly 9.4% of women reported smoking before pregnancy and 7.1% during pregnancy, both at high and low intensity, and smoking rates were higher in the first trimester (7.1%) than in the second (6.1%) or the third (5.7%) trimester. Non-Hispanic White women, women 20–24 years old, and women with less than a high school education were the strongest predictors of smoking anytime during pregnancy. The odds of smoking in early pregnancy at high intensity were 88% lower (aOR 0.12, 95% CI: 0.11, 0.13) for Hispanic women, compared to non-Hispanic White women; 16% higher (aOR 1.16, 95% CI: 1.12, 1.21) for women 20–24 years old and 16% lower (aOR 0.84, 95% CI: 0.80, 0.89) for women ≥35 years old, compared to women 25–29 years old; as well as 13% higher (aOR 1.13, 95% CI: 1.09, 1.18) for women with less than a high school education and 92% lower (aOR 0.08, 95% CI: 0.08, 0.09) for women with a bachelor’s degree or higher, compared to women with a high school diploma.

**Conclusions:**

Despite the high prevalence of high intensity smoking before and during pregnancy, future intervention strategies need to focus on the proportion of low intensity quitters and reducers, who are ready to stop smoking. Continual monitoring of trends in smoking intensity patterns is necessary, including neonatal outcomes over time.

## Plain English summary

Pregnancy offers an opportunity to modify cigarette smoking behavior but the intensity of smoking during pregnancy remains high and cessation is an ongoing challenge. This study examined smoking in the 3 months before pregnancy and during pregnancy (by trimester) and identified women, who gave birth in all 50 states and the District of Columbia in 2016, by smoking intensity status and by race/ethnicity, age and educational level. Nearly 9.5% of women smoked before pregnancy and 7.1% during pregnancy, both at high and low intensity. Non-Hispanic White women, 20–24 years old, and women with less than a high school education were at the highest risk of high intensity smoking in early pregnancy that carries serious adverse effects on fetal growth and newborn outcomes. Quitting before pregnancy is best. Women who enter pregnancy as smokers and do not change their smoking activity should be targeted in early public health cessation interventions.

## Background

In the United States, despite a decline in the overall prevalence of cigarette smoking during the past decade, [1] smoking rates before and during pregnancy have not changed much and disparities remain among some subpopulations of pregnant women and vary markedly across states [2, 3]. Currently, twenty out of every one hundred women 18–44 years old are smokers and some smoke cigarettes in combination with using other forms of tobacco products [4]. A national report for 2014, [5] based on the 95% revised US standard birth certificate, shows that almost 11% (10.9%) of women smoked in the 3 months before pregnancy, three-fourths (75.8%) of these women smoked during pregnancy, and about one- fourth (24.2%) quit smoking before becoming pregnant [5]. The prevalence of smoking in the first and second trimester was higher because about 20% of women quit smoking in the third trimester. Non-Hispanic White women, 20–24 years old, and women with less than a high school education had the highest rates of smoking throughout pregnancy [5].

Historically, smoking patterns among pregnant women mirrored smoking behavior in the general population of women, in terms of rates and downward trends. Years before the United States Surgeon General’s Report on the harmful effects of nicotine on health was published in 1964, [6] the prevalence of smoking in the general population of women continued to rise likely influenced by aggressive marketing of tobacco products to women that began in the 1920s [7–9]. Before 1955, no nationally representative surveys on individual smoking habits were conducted and women were not included in prospective epidemiologic studies [10]. During the peak of the tobacco epidemic most physicians smoked and various tobacco products were advertised in medical journals and exhibited at medical conventions [11, 12]. The fourth stage of the smoking epidemic that began in developed countries in the 1980s was marked with significant reductions in smoking in every social class [13]. While in the US in the 1970’s approximately 40.0% of White and 33.0% of Black women smoked in pregnancy, [14] in 1999 nearly 16% of White and 9.5% of Black women who gave birth reported smoking, [15] and in 2014, 12% of White and 7% of Black women smoked at any time during pregnancy [5]. Also, a remarkable increase in quit rates occurred over time, so that by 1999, the odds that a woman would quit smoking during pregnancy were 51% greater than in 1993 (aOR 1.51; 95% CI:1.08,2.12) and smoking at low intensity before pregnancy strongly predicted quitting in pregnancy [16].

In most developed countries, the overall smoking rates began to fall and the prevalence of smoking during pregnancy has been changing. For instance, in the United Kingdom, the reported rates of cigarette smoking at the time of delivery have declined from 15.1% in 2006–2007 to 12.0% in 2013–2014 [17]. In Australia [18] and in Canada [19] current rates of smoking in pregnancy are comparable to those of the US (i.e. 9–10%) [5]. While in the United States the smoking rate during pregnancy is considerably lower than in Ireland (38.4%), Uruguay (29.7%), and Bulgaria (29.4%), yet it remains higher than the current global average rate of 1.7% [20]. One of the key objectives of Healthy People 2020 is to increase abstinence during pregnancy by 10% to a target rate of 98.6% [21]. Public health-directed tobacco control policies and programs brought about substantial declines in overall smoking rates, probably by an increase in cessation and a reduction in initiation of smoking, [22] however, relatively little is known whether similar reductions occurred among pregnant women and how they influenced perinatal outcomes [23, 24].

Pregnancy outcomes and the future health of newborns are associated not only with prevalence but with smoking intensity status during pregnancy indicating the risk of prenatal exposure. Although higher smoking intensity (measured by the number of cigarettes smoked/day) is shown to be more harmful on the fetus than lower intensity, [25, 26] no level of smoking during pregnancy is safe. The available literature on the prevalence of smoking in pregnancy by intensity status across maternal characteristics is limited. Given unpredictable smoking behavior in pregnancy, [27, 28] it is important to monitor successive birth cohorts for any change in maternal smoking intensity status in association with neonatal outcomes.

The objective of this study is to present the first 100% nationally representative data on the prevalence of cigarette smoking by intensity status in the three months before and during pregnancy (per trimester) according to maternal characteristics, based on the 2016 US National Center for Health Statistics (NCHS) Natality File of Live Births. It is postulated that in the United States the prevalence of smoking at high intensity before and during pregnancy remains high and significant disparities in smoking exist across maternal race/ethnicity, age, and educational attainment in early and late pregnancy.

## Methods

### Study design and data source

This was a cross-sectional study of 3,956,112 live births registered in the 2016 US National Center for Health Statistics (NCHS) Natality File, the first 100% nationally representative data based on the 2003 fully revised birth certificate [29]. As of January 2016, birth certificate revisions were implemented in all 50 states and the District of Columbia providing expanded information on cigarette smoking in the three months before pregnancy and in each trimester of pregnancy, as well as new information on other variables such as the highest educational degree attained, pre-pregnancy weight and height, weight at delivery and usage and month of initiation of prenatal care [30]. After excluding missing data on smoking by trimester (*n* = 79,089) on maternal race, ethnicity (*n* = 36,466) and education (*n* = 51,721), the analytic sample consisted of 3,868,088 live births that occurred at any gestational age.

### Quality of self-reported data

Self-reports imply underreporting and/or nondisclosure of smoking as well as overreporting of cessation, and are prone to bias [31–34]. Almost 25% of pregnant women do not disclose smoking during pregnancy related in part to social stigma [32, 35] and about 42% of women who self-reported abstinence failed biochemical verification in a clinical trial [36]. The highest sensitivity of biochemical assessment was in the 3 months before pregnancy relative to any trimester of pregnancy [37] and the quality of reproductive, maternal and infant health data was reported high when compared with hospital medical records [38, 39].

### Maternal characteristics

Maternal race, ethnicity, age, and educational attainment were the variables of interest in this study and were self-reported on the birth certificate. Race and ethnicity were classified based on the 1997 US Office of Management and Budget (OMB) classification standards [40] and the leading categories in this study were: non-Hispanic White women, non-Hispanic Black women, Hispanic women, and other race/ethnicity groups i.e., non-Hispanic Asian and non-Hispanic American Indian/Alaska Native [41]. Maternal age was categorized as less than 20, 20–24, 25–29, 30–34, or 35 years old and older, and maternal education was categorized as less than high school, high school diploma /GED (General Educational Development) certificate, some college/associate degree, and a bachelor’s degree or higher.

### Smoking status measures

Self-reported smoking on the birth certificate includes the average number of cigarettes (or packs) smoked per day in the 3 months before pregnancy and in each trimester of pregnancy, and smoking anytime during pregnancy is defined as smoking in any of the three trimesters of pregnancy [42]. It was assumed that women who reported smoking in pregnancy were active smokers because nonsmokers would be less likely to report active smoking [43]. In this study women were considered smokers if they reported smoking at least one cigarette per day/trimester and considered non-smokers if they reported no cigarette use in any trimester [44]. Timing of smoking was assessed in the three months before pregnancy and in each trimester in which smoking was reported, and smoking intensity level was assessed by the number of cigarettes smoked per day and categorized as low intensity (1–9 cigarettes/day) and high intensity (≥10 cigarettes/day) [25, 45].

Given complex and unpredictable smoking behavior in pregnancy, [27, 28] standard methodology was advanced in this study to better capture the spectrum of natural maternal variability in smoking behavior across trimesters that indicates the risk of prenatal smoking exposure. The following new seven mutually exclusive smoking intensity status categories were created: (1) *Quitter-Low:* low intensity smoking in early pregnancy only (the first and second trimester only, none in the third trimester); (2) *Quitter-High*: high intensity smoking in early pregnancy only (the first and second trimester only, none in the third trimester); (3) *Maintainer-Low*: low intensity smoking in early pregnancy (first and second trimester) and low intensity smoking in late pregnancy (third trimester); (4) *Maintainer-High:* high intensity smoking in early pregnancy (first and second trimester) and high intensity smoking in late pregnancy (third trimester); (5) *Reducer:* high intensity smoking in early pregnancy (first and second trimester) and low intensity smoking in late pregnancy (third trimester); (6) *Increaser:* low intensity smoking in early pregnancy (first and second trimester) and high intensity smoking in late pregnancy (third trimester); (7) *Nonsmoker:* no cigarette use in any trimester.

### Statistical analyses

Pre-pregnancy was defined as the period in the three months before pregnancy [5]. Pregnancy was divided into three trimesters (stages): the first trimester (months 1–3 or weeks 1–13), the second trimester (months 4–6 or weeks 14–27), and the third trimester (months 7 to delivery or 28 weeks to delivery) [46]. Analyses of maternal smoking in the three months before pregnancy and in each trimester of pregnancy by race/ethnicity, age and educational attainment were performed to estimate prevalence of smoking by intensity status. Next, the distribution of smoking intensity status in early (first and second trimester) and late (third trimester) pregnancy was examined across maternal characteristics. Statistical significance for all tests was defined as *p* < 0.05. Finally, multivariate logistic regression analyses were conducted to assess the odds of smoking at low and at high intensity level in early pregnancy in association with maternal characteristics, adjusted for race/ethnicity, age, and education. Strengths of association were reported as adjusted odds ratios (aORs) and 95% confidence intervals (CIs). This study analyses involved the entire population, so weights were not used. All analyses were performed using SAS version 9.4 (SAS Institute Inc., Cary, NC). This was a secondary analysis of deidentified and publicly available data and the study received an exemption from the University of Maryland Institutional Review Board (IRB). The author has no financial or other conflict of interest.

## Results

Table 1 shows that the overall cigarette smoking rate in the three months before pregnancy was 9.4% (3.1% (95% CI: 3.1, 3.2) at low and 6.3% (95% CI: 6.3, 6.4) at high intensity, and 7.1% (95% CI: 7.0, 7.1) of women reported smoking anytime during pregnancy. Before pregnancy, high intensity smoking (≥10 cigs/day) was most prevalent among non-Hispanic White women, 20–24 years old, and women with a high school diploma (Table 1). During pregnancy, high intensity smoking was more likely in the first trimester (4.2%; 95% CI: 4.1, 4.2) than in the second (3.2%; 95% CI: 3.1, 3.2) or in the third trimester (2.7%; 95% CI:2.6, 2.7), and women with less than a high school diploma/GED certificate smoked mostly at high intensity (Table 1). Also, smoking anytime during pregnancy was most prevalent among non-Hispanic White women, 20–24 years old, and among women with less than a high school education (Table 1). Table 2 shows that in early and in late pregnancy 36.9% (95% CI: 36.6, 37.2) of women continued to smoke at high intensity (*Maintainers-High)* and 28.7% (95% CI: 28.4, 29.0) at low intensity (*Maintainers-Low)*. Maternal predictors of continued smoking at high intensity in early and late pregnancy (*Maintainers-High*) were non-Hispanic White women (41%; 95% CI: 41.2, 41.7) and women who were 35 years and older (42%; 95% CI: 41.2, 42.3), and predictors of smoking at low intensity (*Maintainers-Low*) were non-Hispanic Black women (45%; 95% CI: 44.5, 45.6) and women less than 20 years old (31%; 95% CI: 30.5, 31.9). However, women with less than a high school education were predictors of both high (41%; 95% CI: 40.1, 41.7) (*Maintainers-High*), as well as low intensity smoking (30%; 95% CI: 29.6, 30.3) (*Maintainers-Low*) in early and late pregnancy. By comparison, nearly 12.8% (95% CI: 12.5, 13.1) of low intensity smokers in early pregnancy (*Quitters-Low*) quit spontaneously by the third trimester (late pregnancy), relative to high intensity smokers (7.1%; 95% CI: 6.7, 7.5) (*Quitters-High*). Hispanic women, < 20 years old, and women with a bachelor’s degree or higher who smoked at high or at low intensity were strongest predictors of quitting in early pregnancy (Table 2). Almost 13.8% (95% CI: 13.5, 14.1) of women who reduced their intensity of smoking from early to late pregnancy (*Reducers*) were likely to be non-Hispanic White women, women 20–24 years old, and women with some college education. Less than 1% (0.7%; 95% CI: 0.3, 1.1) of women, who increased their intensity of smoking during pregnancy *(Increasers)* were likely to be non-Hispanic White women and women of other race/ethnicity, aged less than 20 and more than 35, with less than a high school education, with high school diploma/GED certificate and a bachelor’s degree or higher education (Table 2). All results were considered statistically significant at *p* < .05.

**Table 1:** Prevalence of smoking by intensity level before and during pregnancy across maternal characteristics, United States, 2016

SMOKING STATUS % (95% CI)									
	Three months before pregnancy at low intensity (*n* = 121,197)	Three months before pregnancy at high intensity (*n* = 244,473)	Smoking anytime during pregnancy (*n* = 279,063)	First trimester at low intensity(*n* = 112,072)	First trimester at high intensity (*n* = 160,506)	Second trimester at low intensity (*n* = 110,411)	Second trimester at high intensity (*n* = 122,130)	Third trimester at low intensity(*n* = 116,985)	Third trimester at high intensity (*n* = 103,731)
Overall % (95% CI)	3.1 (3.1, 3.2)	6.3 (6.3, 6.4)	7.1 (7.0, 7.1)	2.9 (2.8, 2.9)	4.2 (4.1, 4.2)	2.9 (2.8, 2.9)	3.2 (3.1, 3.2)	3.0 (3.0, 3.1)	2.7 (2.6, 2.7)
Maternal Characteristics									
Race/ethnicity									
Non-Hispanic White	3.6 (3.5, 3.6)	9.8 (9.8, 9.9)	10.5 (10.4, 10.5)	3.7 (3.6, 3.7)	6.6 (6.5, 6.6)	3.8 (3.7, 3.8)	5.1 (5.0, 5.1)	4.1 (4.1, 4.2)	4.4 (4.3, 4.4)
Non-Hispanic Black	4.3 (4.2, 4.4)	3.6 (3.5, 3.6)	6.0 (5.9, 6.0)	3.7 (3.6, 3.7)	2.1 (2.1, 2.2)	3.3 (3.2, 3.3)	1.5 (1.4, 1.5)	3.2 (3.1, 3.2)	1.3 (1.2, 1.3)
Hispanic	1.6 (1.5, 1.6)	1.3 (1.2, 1.3)	1.8 (1.7, 1.8)	1.0 (0.9, 1.0)	0.7 (0.6, 0.7)	0.8 (0.8, 0.9)	0.5 (0.4, 0.5)	0.8 (0.8, 0.9)	0.4 (0.3, 0.4)
Other race/ethnicity ^a^	2.7 (2.6, 2.7)	3.4 (3.4, 3.5)	4.5 (4.5, 4.6)	2.3 (2.2, 2.3)	2.2 (2.1, 2.2)	2.1 (2.0, 2.1)	1.5 (1.5, 1.6)	2.1 (2.1, 2.2)	1.2 (1.2, 1.3)
Age (years)									
< 20	4.4 (4.3, 4.5)	7.1 (7.0, 7.3)	8.5 (8.4, 8.6)	4.0 (3.9, 4.1)	4.2 (4.1, 4.3)	3.7 (3.6, 3.8)	2.9 (2.8, 2.9)	3.8 (3.7, 3.9)	2.3 (2.2, 2.4)
20–24	4.9 (4.8, 4.9)	9.3 (9.2, 9.4)	10.7 (10.7, 10.8)	4.6 (4.5, 4.6)	5.9 (5.9, 6.0)	4.4 (4.4, 4.5)	4.3 (4.3, 4.4)	4.7 (4.6, 4.7)	3.6 (3.5, 3.6)
25–29	3.4 (3.3, 3.4)	7.1 (7.1, 7.2)	8.2 (8.1, 8.2)	3.2 (3.1, 3.2)	4.8 (4.7, 4.8)	3.2 (3.1, 3.2)	3.7 (3.7, 3.8)	3.4 (3.3, 3.4)	3.2 (3.1, 3.2)
30–34	2.2 (2.1, 2.2)	4.7 (4.6, 4.7)	5.2 (5.1, 5.2)	2.0 (1.9, 2.0)	3.1 (3.1, 3.2)	2.0 (1.9, 2.0)	2.5 (2.4. 2.5)	2.1 (2.0, 2.1)	2.1 (2.1, 2.2)
35+	1.8 (1.7, 1.8)	3.8 (3.7, 3.8)	4.3 (4.2, 4.3)	1.6 (1.5, 1.6)	2.6 (2.5, 2.6)	1.6 (1.5, 1.6)	2.1 (2.0, 2.1)	1.7 (1.6, 1.7)	1.8 (1.7, 1.8)
Education									
Less than high school	4.7 (4.6, 4.7)	10.1 (10.0, 10.2)	12.8 (12.8, 12.9)	4.9 (4.9, 5.0)	7.6 (7.6, 7.7)	5.1 (5.1, 5.2)	6.2 (6.1, 6.2)	5.5 (5.4, 5.5)	5.4 (5.3, 5.4)
High School/GED ^b^	4.8 (4.7, 4.8)	10.3 (10.3, 10.4)	11.9 (11.9, 12.0)	4.7 (4.7, 4.8)	7.0 (6.9, 7.0)	4.7 (4.7, 4.8)	5.4 (5.3, 5.4)	5.0 (5.0, 5.1)	4.6 (4.5, 4.6)
Some college/Assoc.	3.6 (3.6, 3.7)	7.1 (7.0, 7.1)	7.6 (7.5, 7.6)	3.2 (3.1, 3.2)	4.2 (4.2, 4.3)	3.0 (2.9, 3.0)	3.0 (3.0, 3.1)	3.2 (3.1, 3.2)	2.5 (2.5, 2.6)
Bachelor’s +	0.8 (0.7, 0.8)	0.9 (0.8, 0.9)	0.8 (0.7, 0.8)	0.4 (0.8, 0.9)	0.4 (0.3, 0.4)	0.3 (0.2, 0.3)	0.2 (0.2, 0.2)	0.3 (0.2, 0.3)	0.2 (0.2, 0.2)

Low intensity smoking: < 10 cigs/day/trimester; High intensity smoking: ≥10 cigs/day/trimester; ^a^ Other race/ethnicity: Asian, American Indian/Alaska Native, Native Hawaiian, Pacific Islander or mixed race; ^b^ GED: General Educational Development; CI: Confidence interval

**Table 2:** Distribution of smoking intensity status in early and late pregnancy according to maternal characteristics, United States, 2016

SMOKING STATUS % (95% CI)							
	TOTAL (*N* = 275,101)	QUITTER-LOW (*n* = 35,161)	QUITTER-HIGH (*n* = 19,577)	MAINTAINER-LOW (*n* = 79,059)	MAINTAINER-HIGH (*n* = 101,540)	REDUCER (*n* = 37,926)	INCREASER (*n* = 1838)
	Smoking in Early or Late pregnancy	Low intensity smoking in Early pregnancy only	High intensity smoking in Early pregnancy only	Low intensity smoking in Early and Low intensity smoking in Late pregnancy	High intensity smoking in Early and High intensity smoking in Late pregnancy	High intensity smoking in Early and Low intensity smoking in Late pregnancy	Low intensity smoking in Early and High intensity smoking in Late pregnancy
Overall % (95% CI)	7.1 (7.0, 7.1)	12.8 (12.5, 13.1)	7.1 (6.7, 7.5)	28.7 (28.4, 29.0)	36.9 (36.6, 37.2)	13.8 (13.5, 14.1)	0.7 (0.3, 1.1)
Maternal Characteristics							
Race/ethnicity							
Non-Hispanic White	76.3 (76.2, 76.5)	10.7 (10.6, 10.9)	7.2 (7.1, 7.3)	25.2 (25.0, 25.4)	41.4 (41.2, 41.7)	14.8 (14.6, 14.9)	0.7 (0.6, 0.7)
Non-Hispanic Black	11.9 (11.8, 12.0)	18.6 (18.2, 19.0)	5.8 (5.5, 6.0)	45.1 (44.5, 45.6)	20.9 (20.4, 21.3)	9.1 (8.8, 9.4)	0.6 (0.5, 0.7)
Hispanic	5.8 (5.7, 5.9)	23.3 (22.7, 24.0)	8.4 (8.0, 8.8)	36.4 (35.7, 37.2)	20.6 (20.0, 21.3)	10.6 (10.2, 11.1)	0.6 (0.5, 0.7)
Other race/ethnicity ^a^	6.0 (5.9, 6.1)	17.3 (16.8, 17.9)	7.2 (6.8, 7.6)	34.5 (33.7, 35.2)	26.8 (26.1, 27.5)	13.5 (13.0, 14.0)	0.7 (0.6, 0.9)
Age (years)							
< 20	6.3 (6.2, 6.4)	18.5 (18.0, 19.1)	8.7 (8.3, 9.1)	31.2 (30.5, 31.9)	26.7 (26.0, 27.4)	14.1 (13.6, 14.6)	0.8 (0.7, 0.9)
20–24	30.4 (30.2, 30.6)	14.5 (14.2, 14.7)	7.5 (7.3, 7.7)	29.6 (29.3, 29.9)	33.0 (32.7, 33.4)	14.7 (14.5, 15.0)	0.6 (0.6. 0.7)
25–29	33.0 (32.8, 33.2)	11.9 (11.7, 12.1)	6.6 (6.5, 6.8)	28.3 (28.0, 28.6)	38.7 (38.4, 40.0)	13.9 (13.6, 14.1)	0.6 (0.6, 0.7)
30–34	20.3 (20.2, 20.5)	10.9 (10.6, 11.2)	6.8 (6.6, 7.0)	28.0 (27.6, 28.4)	40.6 (40.2, 41.0)	13.0 (12.7, 13.3)	0.7 (0.6, 0.8)
35+	10.0 (9.9, 10.1)	10.7 (10.4, 11.1)	7.2 (6.9, 7.5)	27.4 (26.9, 28.0)	41.8 (41.2, 42.3)	12.2 (11.8, 12.6)	0.8 (0.7, 0.9)
Education							
Less than HS	24.6 (24.5, 24.8)	9.4 (9.2, 9.6)	5.3 (5.1, 5.5)	30.0 (29.6, 30.3)	41.4 (40.1, 41.7)	13.3 (13.0, 13.5)	0.70 (0.7, 0.8)
High School/GED ^b^	41.6 (41.4, 41.8)	12.0 (11.8, 12.2)	6.8 (6.7, 7.0)	28.6 (28.3, 28.9)	38.0 (37.7, 38.3)	14.0 (13.8, 14.2)	0.7 (0.6, 0.7)
Some college	30.4 (30.2, 30.5)	15.4 (15.2, 15.7)	8.6 (8.4, 8.8)	27.9 (27.6, 28.2)	33.1 (32.8, 33.4)	14.4 (14.2, 14.7)	0.6 (0.6, 0.7)
Bachelor’s +	3.5 (3.4, 3.5)	23.5 (22.7, 24.4)	11.2 (10.5, 11.8)	29.3 (28.4, 30.2)	25.3 (24.5, 26.2)	10.0 (9.4, 10.6)	0.7 (0.5, 0.9)

Early pregnancy: 1st and 2nd trimester; Late pregnancy: 3rd trimester; Low intensity smoking: < 10 cigs/day/trimester; High intensity smoking: ≥10 cigs/day/trimester; ^a^ Other race/ethnicity: Asian, American Indian/Alaska Native, Native Hawaiian, Pacific Islander or mixed race; ^b^ GED: General Educational Development; CI: Confidence Interval

Results of multivariate logistic regression analyses are displayed in Table 3. Hispanic women had 76% lower odds (aOR 0.24; 95% CI: 0.23, 0.25) of smoking at low intensity (1–9 cigs/day) and 88% lower odds (aOR 0.12; 95% CI: 0.11, 0.13) of smoking at high intensity (≥10 cigs/day) in early pregnancy, compared with non-Hispanic White women. Likewise, women who were 35 years old or older had a 34% lower odds (aOR 0.66; 95% CI: 0.63, 0.69) of smoking at low intensity and a 16% lower odds (aOR 0.84; 95% CI: 0.80, 0.89) of smoking at high intensity in early pregnancy, compared with women 25–29 years old. Finally, women with a bachelor’s degree or higher had 88% lower odds (aOR 0.12, 95% CI: 0.12, 0.13) of smoking at low intensity and 92% lower odds (aOR 0.08; 95% CI: 0.08, 0.09) of smoking at high intensity in early pregnancy, compared to women with a high school diploma/GED certificate (Table 3).

**Table 3:** Adjusted odds ratios of smoking at low and high intensity in early pregnancy stratified by maternal characteristics

Maternal Characteristic	Low-intensity smoking *N* = 35,161 (12.78%)		High-intensity smoking *N* = 19,577 (7.12%)	
Maternal Race/ethnicity	aOR	95% CI	aOR	95% CI
Non-Hispanic White (ref.)	1	1	1	1
Non-Hispanic Black	0.70	0.68, 0.72	0.32	0.30, 0.33
Hispanic	0.24	0.23, 0.25	0.12	0.11, 0.13
Other race/ethnicity ^a^	0.79	0.76, 0.82	0.47	0.45, 0.50
Maternal Age (years)				
< 20	1.22	1.17, 1.27	1.01	0.94, 1.06
20–24	1.26	1.23, 1.30	1.16	1.12, 1.21
25–29 (ref.)	1	1	1	1
30–34	0.76	0.73, 0.78	0.88	0.85, 0.92
35+	0.66	0.63, 0.69	0.84	0.80, 0.89
Maternal Education				
Less than high school	1.05	1.01, 1.08	1.13	1.09, 1.18
High School/GED ^b^ (ref.)	1	1	1	1
Some College	0.79	0.77, 0.81	0.71	0.68, 0.73
Bachelor’s +	0.12	0.12, 0.13	0.08	0.08, 0.09

Early pregnancy: 1st and 2nd trimester; Low intensity smoking < 10 cigs/day/trimester; High intensity smoking ≥10 cigs/day/trimester; ^a^ Other race/ethnicity: Asian, American Indian/Alaska Native, Native Hawaiian, Pacific Islander or mixed race; ^b^ GED: General Educational Development; aOR: adjusted odds ratio; CI: confidence interval; Adjusted for: race/ethnicity, age, and educational attainment

## Discussion

This is the first 100% nationally representative study, based on the 2016 US National Center for Health Statistics (NCHS) Natality File of Live Births, to report data on the prevalence of cigarette smoking by intensity status before and during pregnancy across selected maternal characteristics, as predictors. Unique to this study, not previously reported in the scientific literature, was examining trimester-specific smoking intensity status in a broader context of natural maternal variability in smoking patterns. Moreover, patterns in smoking intensity in early pregnancy are of interest in this study not only because women who enter pregnancy as high intensity smokers continue to smoke at high intensity, [5] but also because prenatal exposure in early months of pregnancy has significant adverse impact on placental [47] and embryonal/fetal growth, [48–50] increasing the risk of miscarriage, [51] preterm birth and low birthweight [25, 52]. This study adds to evidence that in the United States the prevalence of high intensity smoking before and during pregnancy remains high and significant disparities in smoking by intensity status exist in early (first and second trimester) and late (third trimester) pregnancy among subpopulations of women. Yet, a large proportion of women who quit or reduce their smoking intensity in early pregnancy show motivation and greater readiness to stop smoking during transition into pregnancy and deserve definite support in targeted interventions.

The US National Vital Statistic Report from 2014 [5] revealed that the smoking rate anytime during pregnancy was 8.4%, compared with 7.1% in this study, and it was most prevalent among non-Hispanic White women, aged 20–24, and less educated women. However, there was no information in the report on smoking intensity status during pregnancy. Analyses by Kurti et al., [53] based on the 2013–2014 Population Assessment of Tobacco and Health (PATH) data from the US, showed that the prevalence of traditional cigarette smoking among pregnant women aged ≥18 was high, however, the authors did not consider smoking intensity patterns in their assessment. In the current study, an estimated 65% of women (36.9% at high and 28.7% at low intensity) continued smoking into late pregnancy (third trimester), compared to about 50% reported in the 2000–2010 PRAMS (Pregnancy Risk Assessment Monitoring System) study, based on the US birth certificate data from 40 sites [2]. A study by Alshaarawy & Anthony [54], based on data from the 2002–2009 US National Survey on Drug Use and Health (NSDUH), used a month-specific approach to assess smoking prevalence in pregnancy and found the highest prevalence rates between months 1–6, corresponding to early pregnancy (1st and 2nd trimester) in the current study. The investigators did not report on the prevalence of smoking by intensity status.

Numerous factors have been associated with smoking and quitting during pregnancy and observed disparities in smoking prevalence are likely multifactorial [33, 55–59]. When trying to answer questions “why do women continue to smoke in pregnancy?” [59, 60] and “why do disparities exist in prenatal smoking?”, [3] research has persistently linked smoking and quitting rates in pregnancy to women’s race/ethnicity, age, educational level and socioeconomic status, as the strongest predictors of smoking. Racial or ethnic differences in smoking could be partly explained by sociocultural influences relevant to the acceptability of tobacco use [58] and disparities by age and education are attributable to individual life circumstances, levels of addictive behavior, and understanding of harms related to tobacco use [3, 55, 60–63]. Non-Hispanic White women, 20–24 years old, women with a high school diploma/GED certificate were mainly high intensity smokers in pre-pregnancy and major predictors of smoking during pregnancy. At the same time, predictors of spontaneous quitting during pregnancy were low intensity smokers of Hispanic ethnicity, age < 20, with a higher level of education, which is consistent with other studies [16, 64, 65]. College-educated women were not only less likely to smoke prior to pregnancy, but also significantly more likely to quit (OR 1.74; 95% CI: 1.44, 2.10) during pregnancy [16].

It is of particular concern that high smoking intensity is so prevalent across trimesters of pregnancy and smoking cessation remains a challenge. The risk of smoking in early pregnancy at high intensity was 16% higher among women 20–24 years old but 16% lower among women 35 years old and older. Higher intensity of smoking later in pregnancy has been attributed to increasing stress of advancing pregnancy and/or guilt of not being able to quit smoking [66]. Nevertheless, a majority of women are motivated to quit or reduce their smoking upon entering pregnancy and smoking rates in late pregnancy have commonly been lower than the overall reported daily smoking rates in the general population of women [67]. Light smokers may quit spontaneously before or after the first prenatal visit, but heavy smokers find it more difficult to quit [68, 69]. The results on smoking reduction during pregnancy in this study reinforced findings from other studies [70–72]. A clear trend in smoking has been observed among more educated women (i.e., at least a bachelor’s degree), who smoked considerably less both before and during pregnancy, which is consistent with a study based on PRAMS (in ten states) [16]. Women with at least a bachelor’s degree had a 92% lower risk (aOR 0.08, 95% CI: 0.08, 0.09) of high intensity smoking in early pregnancy than less educated women. These findings are also supported by a study on educational disadvantage as a predictor of smoking status during pregnancy [73]. It is relevant to note that underreporting of smoking [74] and self-reported quit rates have been considerably higher among college-educated (i.e., a bachelor’s degree and higher) than among non-college-educated women [73, 75]. New tobacco products including electronic cigarettes and hookah are becoming popular among young women adding to the increased risk of prenatal smoking exposure [4, 76, 77].

Research indicates that complete smoking cessation in early pregnancy is most effective in decreasing the risk of adverse newborn outcomes, including preterm birth and low birthweight [25, 26, 48, 49, 78]. Women are becoming increasingly aware that smoking during pregnancy is unsafe and acknowledge the potential harms of smoking, but they may not fully recognize the seriousness of smoking effects on the health of a child and believe that it is acceptable to smoke during pregnancy [79–81]. Therefore, cessation interventions implemented before conception are best, but support offered anytime during pregnancy to quitters and reducers is strongly warranted to maintain cessation and prevent relapse. Current evidence is insufficient regarding smoking cessation treatment options (e.g., bupropion, varenicline) that could be recommended to pregnant women, because of the concern for teratogenicity [82].

### Strengths and limitations

This study benefited from several strengths. The main strength was availability of the first 100% nationally representative data of live births with numerous variables, thus making the findings of this study generalizable to the entire US population. New smoking intensity status categories were created in this study to capture the spectrum of natural maternal variability in smoking patterns during pregnancy. There are also some limitations of this study. An inherent limitation comes from its cross-sectional design. Self-reports are prone to underreporting and nondisclosure of smoking, as well as overreporting of cessation that could introduce bias [33, 34]. No information on non-daily smoking (< 1 cigarette/day) in pregnancy is available on the birth certificate. Finally, while there is a higher risk of miscarriage among women who smoke at high intensity in early pregnancy, [51] the current study uses the US standard certificate of live birth that includes no information on miscarriage or stillbirth.

## Conclusions

Based on the presented evidence, disparities in the prevalence of smoking by intensity status exist among subpopulations of pregnant women in the United States defined by race/ethnicity, age, and educational attainment. These study findings have implications for planning intervention strategies in preconception or early in pregnancy. While the majority of women who enter pregnancy as smokers continue to smoke at high intensity, a large proportion of low intensity smokers show greater motivation and readiness to quit or reduce their smoking level. These women deserve support in targeted interventions to maintain cessation and prevent relapse. Continual monitoring of trends in the prevalence of smoking by intensity status before and during pregnancy, including any changes in patterns that also reflect neonatal health outcomes is necessary.

## Data Availability

The dataset analyzed during the current study is publicly available from the National Center for Health Statistics (NCHS) Vital Statistics Natality Birth Data and can be accessed through the National Bureau of Economic Research website: http://www.nber.org/data/vital-statistics-natality-data.html.

## Abbreviations

aOR: Adjusted odds ratio
CI: Confidence interval
GED: General educational development
IRB: Institutional Review Board
NCHS: National Center for Health Statistics System
NSDUH: National Survey on Drug Use and Health
OMB: Office of Management and Budget
PATH: Population Assessment of Tobacco and Health
PRAMS: Pregnancy Risk Assessment Monitoring System
